# Establishing MS2-MCP-based single-molecule RNA visualization in *Schizosaccharomyces pombe*

**DOI:** 10.64898/2026.03.09.710516

**Published:** 2026-03-09

**Authors:** Douglas E. Weidemann, Sarah C. Turner, Silke Hauf

**Affiliations:** Department of Biological Sciences, Virginia Tech, Blacksburg, VA 24061, USA; Fralin Life Sciences Institute, Virginia Tech, Blacksburg, VA 24061, USA

**Keywords:** single-molecule RNA imaging, MS2-MCP system, fission yeast, RNA dynamics

## Abstract

Single-molecule RNA imaging using the MS2–MCP system has transformed the study of RNA biology across model organisms. However, this technology has remained unavailable for fission yeast *(Schizosaccharomyces pombe)*, even though fission yeast is a central model for eukaryotic gene expression. Achieving single-molecule sensitivity requires identifying a narrow optimum where RNA labels are sufficiently bright while background fluorescence remains minimal. We have now accomplished this for *S. pombe* by systematically optimizing MCP expression and localization—screening a panel of constitutive *S. pombe* promoters and evaluating combinations of nuclear localization and export signals (NLSs and NESs). The resulting, successful constructs use tandem StayGold as the MCP fluorescent tag, taking advantage of its superior photostability. Together with optimized vectors for MS2 stem-loop tagging of endogenous transcripts, these tools enable single-molecule RNA imaging in fission yeast, opening the door to quantitative analyses of RNA dynamics in this core genetic model.

## Introduction

**Figure 1.**
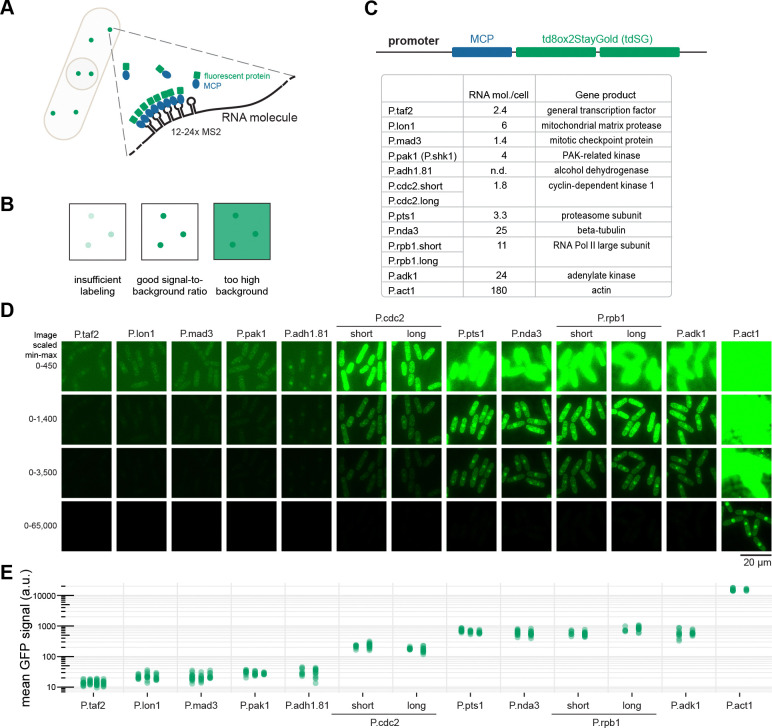
Screening constitutive *S. pombe* promoters to establish RNA imaging with MS2-MCP. **(A)** RNAs are tagged with stem-loops from the bacteriophage MS2. Dimers of MS2 coat protein (MCP) bind to each stem-loop structure. Tagging of MCP with a fluorescent protein allows for visualization of the RNA. **(B)** RNA single molecule imaging requires a high enough MCP-fluorescent protein concentration to efficiently label the RNAs, but low enough concentration to not create a high background signal. **(C)** MCP was tagged with tandem StayGold (tdSG), a highly bleaching-resistant green fluorescent protein and expressed under different promoter sequences from the *S. pombe ura4* locus. To optimize the signal-to-background ratio, 11 *S. pombe* promoters were tested. The promoters are expected to be “housekeeping” or stably expressed based on the function of their gene product or have been reported as such in the literature. The estimates of RNA molecules per cell for each respective gene are from Marguerat et al., Cell 2012. For P.cdc2 and P.rpb1 both a shorter and a longer sequence upstream of the gene’s start codon was tested. **(D)** Example images from strains expressing *mad2*-24xMS2 and MCP-NLS-tdSG from the indicated promoters. Image acquisition conditions were the same; each field of view is shown using four different scaling settings in order to capture the breadth of signal intensities. **(E)** The mean tdSG signal intensity in single cells was quantified from at least two different images for each strain (between 5 and 16 cells per image); dots are individual cells. Note that data are displayed on a logarithmic scale.

The visualization of RNA molecules in live cells provides critical insights into their spatial and temporal dynamics (Gerber et al., 2023; Le et al., 2022; Tutucci et al., 2018). Such visualization has been achieved by repurposing RNA stem-loop-forming sequences from bacteriophages (Bertrand et al., 1998). The first, and still most widely used, version comes from the phage MS2. RNAs that carry MS2 stem-loops can be visualized with fluorescently tagged MS2 coat protein (MCP) (Fig. 1A). This system has enabled imaging of single RNA molecules with high spatial and temporal resolution and has, for example, led to key discoveries on the molecular basis of transcription bursts (Chubb et al., 2006; Donovan et al., 2019; Fukaya et al., 2016; Golding et al., 2005; Patel et al., 2023), splicing (Brody et al., 2011; Coulon et al., 2014; Martin et al., 2013; Schmidt et al., 2011), RNA nuclear export dynamics (Grünwald and Singer, 2010; Mor et al., 2010), RNA localization (Bertrand et al., 1998; Fusco et al., 2003), translation (Halstead et al., 2015; Morisaki et al., 2016; Pichon et al., 2016; Wu et al., 2016) and RNA degradation (Horvathova et al., 2017).

Fission yeast (*Schizosaccharomyces pombe*) is a widely used model organism in molecular biology, including RNA biology (Hoffman et al., 2015; Vyas et al., 2021). Research using *S. pombe* has uncovered fundamental mechanisms of transcription, splicing, nuclear and cytoplasmic RNA decay, and RNA-mediated mechanisms of heterochromatin formation (Fair and Pleiss, 2017; Hirota et al., 2008; Lee et al., 2013; Schramke et al., 2005; Sugiyama and Sugioka‐Sugiyama, 2011; Ukleja et al., 2016; Vo et al., 2019; Volpe et al., 2002; Yan et al., 2015). However, RNA imaging with single-molecule sensitivity has not yet been achieved in *S. pombe*. A few *S. pombe* RNAs have been labelled fluorescently using MS2-MCP, but only to visualize bulk nuclear versus cytoplasmic localizations (Carnahan et al., 2005) or an abundantly transcribed RNA at its transcription site (Shichino et al., 2014).

Critical for single-molecule sensitivity is the expression level and localization of MCP. Expression that is too weak does not yield enough signal from each tagged RNA molecule, whereas overly high expression floods the cell with unbound fluorescent MCP, impairing the visualization of labelled RNA molecules against the high background (Fig. 1B). Screening a broad range of constitutive *S. pombe* promoters and several localization signal combinations, we have now identified MCP constructs suitable for single-molecule RNA visualization in *S. pombe*. These constructs use the highly bleaching-resistant green fluorescent protein StayGold fused to MCP (Ando et al., 2023; Hirano et al., 2022), thus making use of the latest advancements in fluorescent protein technology.

## Results

### Identification of constitutive *S. pombe* promoters suitable for single-molecule MS2-MCP imaging

To optimize the expression of MCP, we selected constitutive *S. pombe* promoters based on gene function (*pts1, nda3*), known low-noise expression (*mad3*, *rpb1* (Weidemann et al., 2023)), stable expression across stress conditions (*cdc2, taf2, lon1, adk1* (Thodberg et al., 2018)), or their prior use in expression vectors (*adh1.81, pak1, act1* (Chen et al., 2017; Kiriya et al., 2017; Vještica et al., 2019; Yokobayashi and Watanabe, 2005)) (Fig. 1C). These promoters span a range of native mRNA expression levels, from around 1 to 180 mRNA molecules per cell (Marguerat et al., 2012). As promoter region, we used between 214 and 913 base pairs upstream of the respective start codon, encompassing both the promoter and the 5’UTR of each gene. Using these promoter regions, we expressed MCP fused to tandem StayGold (td8ox2StayGold, tdSG (Ando et al., 2023)) (Fig. 1D,E). StayGold was chosen due to its superior photostability compared to other fluorescent proteins (Ando et al., 2023; Hirano et al., 2022). We observed four classes of fluorescence intensities, partly, but not perfectly, scaling with the reported RNA numbers: low *(taf2, lon1, mad3, pak1, adh1.81)*, medium *(cdc2)*, high *(pts1, nda3, rpb1, adk1),* and very high expression *(act1)* (Fig. 1E, note the logarithmic scale). Monomeric StayGold (Ando et al., 2023), tested as an alternative to tdSG, yielded signals much weaker than half the tdSG signal, was undetectable with weak promoters, and therefore was not pursued further (Fig. S1).

**Figure 2.**
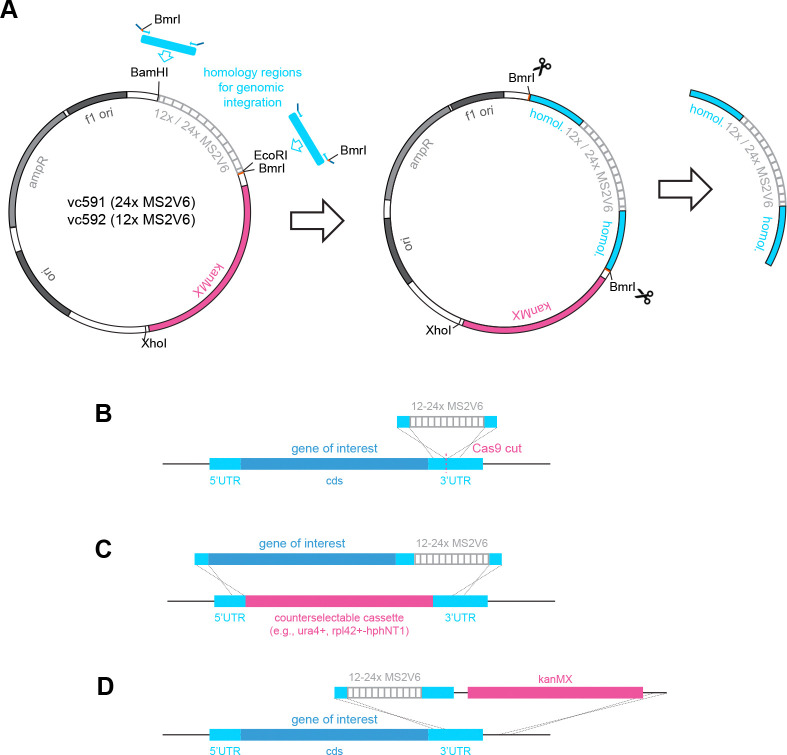
Vectors for attaching homology regions to MS2V6 repeats. **(A)** Vectors to append homology regions upstream and downstream of the MS2V6 repeats; vc591 (24x MS2V6) and vc592 (12x MS2V6) are slightly modified versions of Addgene plasmids # 104393 and # 104392 (Tutucci et al., Nat Methods 2017). An EcoRI site was added downstream of the MS2V6 repeats. Homology regions can be integrated at the BamHI, EcoRI, and XhoI sites. **(B,C,D)** Strategies to integrate the 12–24x MS2V6 repeats into the genome: (B) CRISPR/Cas9-facilitated integration without resistance gene; (C) replacement of a counterselectable cassette, e.g. when working with a non-essential gene; (D) integration using the kanMX resistance gene present on the vector.

To test the feasibility of imaging single RNA molecules, we replaced the wild-type *mad2* gene (a component of the spindle assembly checkpoint (McAinsh and Kops, 2023)) with a version containing 24 MS2 stem-loops in its 3’UTR (*mad2*-24xMS2). By single-molecule RNA fluorescence in situ hybridization (smFISH), the *mad2* gene shows between 0 and 8 mRNA molecules per cell (mean ~ 3) (Esposito et al., 2022; Weidemann et al., 2023). We used the MS2V6 variant for tagging, which has slightly weakened MCP-binding and a longer linker between stem-loops to avoid an artificial stabilization of the MS2 fragment (Tutucci et al., 2017). To facilitate endogenous tagging, we slightly modified existing 12xMS2V6 and 24xMS2V6 vectors (Fig. 2A). The original vectors were designed as PCR templates to attach homology regions to the MS2 repeats; however, PCR across repeat regions often requires optimization (Hommelsheim et al., 2014). We therefore opted for a strategy where homology regions can be cloned into the vector upstream and downstream of the repeats, and the piece to be transformed into yeast is excised using Type IIS restriction enzymes (Fig. 2, S2). The donor DNA containing MS2 tags can be introduced into the genome in a scarless manner by CRISPR/Cas9-mediated homologous recombination (Fig. 2B) or by replacement of a counterselectable cassette (Fig. 2C). Alternatively, the kanamycin(G418)- resistance on the vector can be used (Fig. 2D).

**Figure 3.**
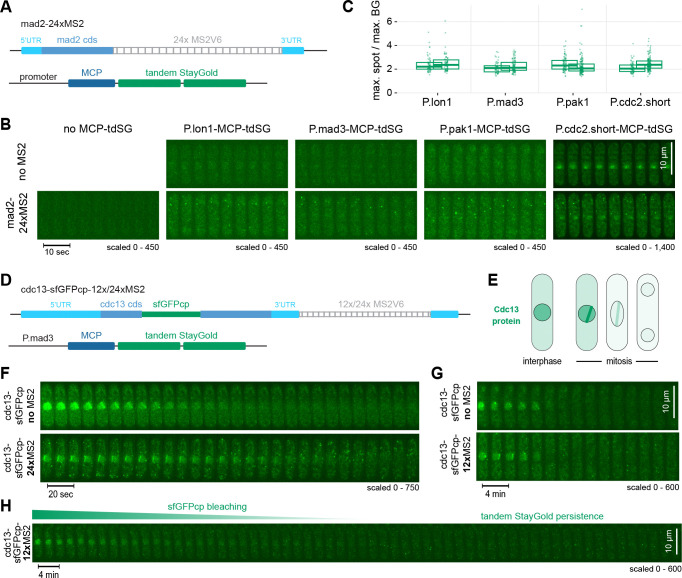
Visualization of single RNA molecules with MCP-tdSG expressed from different promoters. **(A)** The *mad2* gene was tagged in the 3’UTR with 24x MS2V6, and MCP-tdSG was expressed from different promoters. **(B)** Short kymographs from live-cell imaging; longer sequences are shown in Fig. S3. For each MCP-tdSG construct, a strain without integration of MS2 repeats is shown as control. Far left: strain expressing *mad2*-24xMS2, but no MCP-tdSG. Images are maximum intensity projections of the Z-stack. Note the different scaling setting for P.cdc2.short-MCP-tdSG. **(C)** Quantification of the maximum spot intensity over maximum background intensity. Two replicates per strain, 56–162 spots per replicate. Points: individual spots; box plot: summary statistics (center line, median; box boundaries, 1st and 3rd quartiles). **(D)** The *cdc13* gene was internally tagged with circularly permuted superfolder GFP (sfGFPcp) and 12x or 24x MS2V6 cassettes were inserted in the 3’UTR. The construct was expressed under endogenous *cdc13* regulatory sequences (promoter, terminator) from the *leu1* locus. MCP-tdSG was expressed from the *mad3* promoter. **(E)** Schematic illustrating Cdc13 protein localization. Cdc13 accumulates during interphase and is strongly enriched in the nucleus. Cdc13 localizes to spindle pole bodies and the spindle during early mitosis and becomes degraded at the metaphase-to-anaphase transition. **(F,G)** Kymographs from live-cell imaging of cells undergoing mitosis; *cdc13*-sfGFP tagged with either 24x MS2V6 (F) or 12x MS2V6 (G). A strain without MS2 repeats is shown as control. Images were recorded every 5 sec (F) or 12 sec (G); every second image (every 10 sec, F) or every 10^th^ image (every 2 min, G) is shown. Images are maximum intensity projections of the Z-stack. **(H)** Similar to (G), but showing an interphase cell; loss of the nuclear sfGFPcp signal is due to photobleaching, not degradation.

Using the combination of *mad2–24xMS2* and MCP-tdSG expressed from different promoters, we observed clear dot-like signals in the cytoplasm using the *lon1*, *mad3*, *pak1*, and *cdc2* promoters (Fig. 3A,B, S3). The abundance and movement of these dots are consistent with the expected behavior of individual mRNA molecules (Fig. 3B, S3, Supplemental Movies S1-S4). Such signals were not observed when MCP-tdSG was expressed in the absence of an MS2 tag (Fig. 3B, Supplemental Movies S1-S4). As expected, promoters that were too weak *(taf2)* or too strong (*pts1* or stronger) failed to produce clearly discernible RNA signals (Fig. S3). Similar ratios of RNA signals over cytoplasmic background were obtained with MCP-tdSG expressed from the *lon1*, *mad3*, *pak1*, and *cdc2* promoters, despite the stronger MCP-tdSG expression from the *cdc2* promoter (Fig. 3C, S4), indicating that the MS2 stem-loops are not saturated with MCP when using weaker promoters.

To test a second gene with higher mRNA concentration, we tagged the cyclin *cdc13* with MS2 stem-loops (Fig. 3D). Cdc13 mRNA numbers per cell determined by smFISH range from around 10 to 35 (mean ~ 20) (Bashir et al., 2023; Weidemann et al., 2023). In addition to the MS2 tag in the 3’UTR, the *cdc13* gene was internally tagged with circularly permuted, superfolder GFP (sfGFPcp), so that Cdc13-sfGFPcp protein could be visualized in addition to *cdc13* mRNA. Because the Cdc13 protein enriches strongly in the nucleus (Alfa et al., 1989; Decottignies et al., 2001), the Cdc13-sfGFPcp protein signal is expected to only contribute minimally to background in the cytoplasm (Fig. 3E). With MCP-tdSG expressed from the *mad3* promoter, both Cdc13 protein, which is degraded in mitosis, and *cdc13* mRNA could be tracked (Fig. 3F,G, S5). No obvious difference in Cdc13-sfGFPcp protein signals was observed with or without the MS2 tags, suggesting that the mRNA with MS2 stem-loops remained functional. Dot-like signals in the cytoplasm, consistent with single mRNA molecules, were observed both with a 12x and 24xMS2 tag, but not when *cdc13* was expressed without MS2 tag (Fig. 3F,G, S5, Supplemental Movies S5,S6). MCP-tdSG dot-like signals in the cytoplasm could still be monitored once the Cdc13-sfGFPcp nuclear signal had bleached (Fig. 3H), consistent with the expected bleaching-resistance of tdSG (Ando et al., 2023; Hirano et al., 2022).

Taken together, we have identified several constitutive promoters that allow for single-molecule mRNA imaging in *S. pombe* using MS2 tags and MCP-tdSG. The signals are sufficiently prominent to be observable even with some additional green fluorescent protein background, and tdSG confers considerable bleaching resistance.

### Visualization of cytoplasmic mRNAs benefits from a weak NLS or a combination of NLS and NES

**Figure 4.**
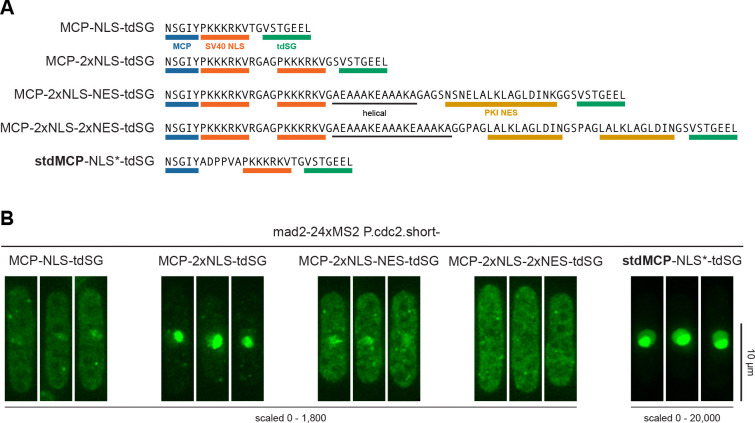
Spatial distribution of MCP-tdSG can be tuned by different combinations of NLS and NES sequences. **(A)** Overview of NLS and NES combinations. NLS and NLS* differ by the linker sequence between MCP and the SV40 NLS. **(B)** Example images from strains expressing *mad2*-24xMS2 and the indicated MCP-tdSG or stdMCP-tdSG constructs. MCP constructs were expressed from the *cdc2* promoter. See Fig. S6 for the same constructs expressed from the *mad3* promoter. All images were recorded with the same exposure conditions; maximum intensity projections of Z-stacks are shown.

MCP-fluorescent protein fusion constructs often include nuclear localization sequences (NLSs) to minimize background of unbound MCP in the cytoplasm, although the argument has also been made that omitting an NLS may be beneficial (Tocchini and Mango, 2024). Our initial MCP-tdSG version included an SV40 NLS with minimal surrounding sequences (Fig. 4A). Using this construct, MCP was visible in the cytoplasm, and the level of nuclear enrichment varied with the promoter and 5’UTR used (Fig. S3). With many of the promoters, MCP-tdSG was further enriched in the nucleolus (Fig. S3). Expression of a tandem MCP construct (stdMCP (Wu et al., 2015)) with the same SV40 NLS, but a longer linker upstream of the NLS (stdMCP-NLS*-tdSG) yielded a much stronger nuclear enrichment—so strong that it became inefficient in labelling cytoplasmic mRNAs (Fig. 4, S6C). Shortening the linker between MCP and NLS increased the fraction of stdMCP-tdSG in the cytoplasm again (Fig. S6C), indicating that the efficiency of the SV40 NLS is compromised by its close vicinity to MCP, but that this may be beneficial for cytoplasmic mRNA imaging. We additionally tested tandem NLSs with either one or two PKI nuclear export signals (NESs) (Fig. 4A).

Addition of a second NLS strongly increased the nuclear enrichment, similar to the single NLS with longer linker, and also decreased the intensity of RNA signals in the cytoplasm (Fig. 4B). Addition of one or two NESs progressively decreased the nuclear and increased the cytoplasmic signal, bringing back cytoplasmic RNA labelling (Fig. 4B). Thus, the nucleocytoplasmic ratio is well tuneable by different NLS and NES combinations. Expression of these constructs from two different promoters *(P.cdc2* and *P.mad3)* yielded qualitatively similar results (Fig. S6A,B).

**Figure 5.**
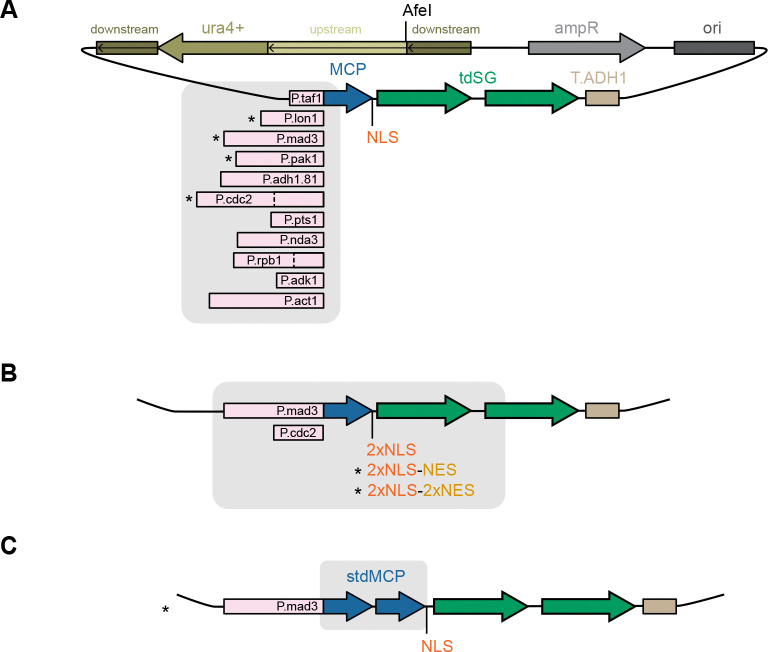
MCP-tdSG vectors for *S. pombe* expression. All vectors are derivatives of pUra4AfeI (Vjestica et al., 2019) and can be integrated at the *ura4* locus after linearization of the vector with AfeI. Vectors marked with an asterisk are likely to be the most useful and will be deposited at Addgene and RIKEN. Other vectors are available upon request. **(A)** Vectors for expression of MCP-NLS-tdSG from different constitutive promoters. The dashed lines for P.cdc2 and P.rpb1 indicate the length of the short promoter versions. **(B)** Vectors for expression of MCP-tdSG with different NLS/NES combinations from either the *mad3* or the short *cdc2* promoter. **(C)** Vector for expression of synonymized tandem MCP (stdMCP, Wu et al., 2015), tagged with NLS and tdSG.

In summary, we have identified conditions for single-molecule mRNA imaging in *S. pombe*. We provide a range of integration vectors for expression of MCP-tdSG that can be paired with MS2-tagged genes (Fig. 5). These vectors use the pUra4AfeI backbone (Vještica et al., 2019) for stable integration into the *S. pombe ura4* locus. These tools now set the stage for single-molecule-based exploration of RNA biology using *S. pombe*.

## Discussion

The MS2-MCP system is widely used for single-molecule RNA live-cell imaging. The absence of its implementation for the model organism *S. pombe* was a notable gap. Here, we close this gap by establishing *S. pombe* expression vectors for MCP fused to bleaching-resistant tandem StayGold that yield cytoplasmic concentrations suitable for MS2 imaging (Fig. 5). By combining MCP-tdSG with several NLS/NES combinations, we provide further flexibility in tuning the nuclear and cytoplasmic signals (Fig. 4,5). We also modified available MS2 vectors (Tutucci et al., 2017) to facilitate MS2 tagging (Fig. 2). Collectively, these tools now enable the exploration of RNA life cycle dynamics and RNA localization with single-molecule precision in *S. pombe* cells. Our data also provide insights into the expression strengths achievable with different constitutive promoters (Fig. 1), which may be informative for other applications; however, we caution that expression strength is further modulated by the coding sequence. For example, in our experiments, the tandem MCP (stdMCP) construct yielded higher concentrations from the same promoter than the single-copy MCP (Fig. S6).

While the constructs we provide here serve as an excellent starting point, further adjustments are possible. On the MCP side, tandem MCP has been reported to provide more consistent signals than single-copy MCP, presumably because MCP acts as a dimer and the tandem construct is poised for dimerization (Wu et al., 2012; Wu et al., 2015). We observed that expression tandem stdMCP-tdSG resulted in an overall stronger signal and stronger nuclear enrichment than tdMCP-tdSG (Fig. S6). Its usefulness can likely be improved by employing a weaker promoter and introducing an NES sequence. Furthermore, it may be possible to increase the signal-over-background ratio by using a recently developed version of MCP that is unstable unless bound to an MS2 stem-loop (Kuffner et al., 2025).

On the MS2 side, it is important to note that repetitive stem-loop tags can alter RNA physiology (Garcia and Parker, 2015; Garcia and Parker, 2016; Haimovich et al., 2016; Heinrich et al., 2016; Li et al., 2022; Tocchini and Mango, 2024; Tutucci et al., 2017). Thus, depending on the application, MS2 tags may require further optimization. For instance, MS2-tagging can lead to RNA mislocalization (Tocchini and Mango, 2024), and MS2 stem-loops may persist in cells after the remainder of the tagged mRNA has been degraded (Garcia and Parker, 2015). These known challenges have prompted the optimization of MS2 tags for linker length, nucleotide composition, and MCP-binding strength (Katz et al., 2021; Tocchini and Mango, 2024; Tutucci et al., 2017; Wu et al., 2015). Here, we have used one of these optimized sequences, MS2V6 (Tutucci et al., 2017), but other optimized sequences may offer further benefits. For example, a version without stop codons will be beneficial for 5’ tagging (Halstead et al., 2015), and versions with shortened linkers may minimize the targeting of tagged mRNAs by nonsense-mediated decay (NMD) (Tocchini and Mango, 2024).

The MS2-MCP system is not the only bacteriophage-derived system used for RNA tagging. The orthogonal PP7-PCP system is similarly popular (Gerber et al., 2023; Le et al., 2022; Tutucci et al., 2018) . The insights gained here from testing different promoters and NLS/NES combinations will be transferable to this system, which further widens the opportunities to monitor and manipulate RNAs in *S. pombe*.

## Materials and Methods

### *S. pomb*e strains

All *S. pombe* strains are listed in Table S1. The *mad2* gene was tagged with a non-fluorescent (Y66L) mutant of EGFP at its C-terminus and with 24xMS2V6 immediately after the stop codon. The tagged *mad2* gene was integrated at the endogenous locus by first deleting the *mad2* coding sequence with a counterselectable *rpl42+/hygR* cassette in an *rpl42*-sP56Q background (Roguev et al., 2007) and then replacing the counterselectable cassette with *mad2*-darkEGFP-24xMS2V6 by selecting first for cycloheximide resistance and then for hygromycin sensitivity. The *cdc13* gene was tagged internally between amino acids S177 and V178 with circularly permuted superfolder GFP (sfGFPcp) (Kamenz et al., 2015) and after nucleotide 205 or 332 of the 3’UTR with 12xMS2V6 or 24xMSV6. The constructs were expressed from a pDUAL vector (Matsuyama et al., 2004) integrated at the exogenous *leu1* locus. No differences in functionality were observed between the integration sites in the 3’UTR. To construct the pDUAL vector, homology regions were first cloned into the 12xMS2V6 and 24xMS2V6 vectors, and a BamHI/XbaI fragment containing the partial coding sequence for *cdc13*-sfGFPcp and the 3’UTR with MS2 repeats was then cloned into a BamHI/SpeI-digested pDUAL vector containing cdc13-sfGFPcp expressed from the *cdc13* promoter. MCP-tdSG constructs were integrated by linearizing the corresponding pUra4AfeI vector with AfeI and transforming it into a *ura4+*-deleted strain (*ura4-D18*). Integration of the vector restores *ura4+*.

### Vectors

All vectors are listed in Table S2. Promoter sequences were amplified from the *S. pombe* genome, except for P.adh1.81, which was from the pRAD81 plasmid (gift from Yoshinori Watanabe). MCP-NLS was from pET296-YcpLac111 CYC1p-MCP-NLS-2xyeGFP (Addgene plasmid # 104394; gift from Robert Singer and Evelina Tutucci); stdMCP-NLS* was from pUbC-nls-ha-stdMCP-stdGFPx (Addgene plasmid # 98916; gift from Robert Singer); td8ox2StayGold was from pBS Coupler1/td8ox2StayGold (RIKEN MDB20227) and mStayGold from pRSETB/mStayGold (RIKEN MDB20214), both by Miyawaki et al. (Ando et al., 2023) and provided by the RIKEN BRC through the National BioResource Project of the MEXT, Japan. The ADH1 terminator from *S. cerevisiae* (*Scer\T.ADH1*) is the same sequence used in the *S. pombe* pFA6a expression vectors (Bähler et al., 1998). Additional NLS and NES sequences were inserted by digest and Gibson assembly with a synthetic fragment.

The vectors containing 12x MSV6 (pET251-pUC 12xMS2V6 Loxp KANr Loxp, Addgene plasmid # 104392) and 24x MS2V6 cassettes (pET264-pUC 24xMS2V6 Loxp KANr Loxp, Addgene plasmid # 104393) were a gift from Robert Singer and Evelina Tutucci (Tutucci et al., 2017). They were modified to incorporate an EcoRI site downstream of the MS2 cassette by BglII digest and Gibson assembly with a synthetic fragment.

### Live-cell microscopy

Imaging was performed on a DeltaVision widefield microscope, using an Olympus 60×/1.42 Plan APO oil objective, 461–489 nm LED illumination, a 525/48 nm GFP/FITC emission filter, a PCO edge sCMOS camera, and an environmental chamber to keep the temperature at 30 °C. Z-stacks were recorded every 5, 6, or 12 sec, as indicated, and Z-sections were spaced by 0.3 to 0.5 μm over a distance of 3.9 to 5 μm. Imaging conditions were kept the same for samples and corresponding controls. Using these conditions, autofluorescence bleached in the first few frames (not shown). Images were deconvolved using SoftWoRx software with three cycles of the ratio method (conservative), noise filtering set to high (300 nm), without corrections, and with a camera intensity offset of 0.

### Whole-cell fluorescence quantification

Cells were segmented based on the brightfield image using YeaZ (Dietler et al., 2020). Quantification was performed on average intensity projections of the Z-stack. To subtract background, the mode of signal measured outside of cells in an image was subtracted from the mean signal obtained from each cell.

### Spot fluorescence quantification

The intensity of RNA spots was measured on a maximum intensity projection of the Z-stack. A region of interest was placed manually on a spot and another region of interest in the cytoplasm of the same cell. The maximum intensity measured for the spot was divided by the maximum intensity measured in the cytoplasm.

## Supplementary Material

Supplement 1

Supplement 2

Supplement 3

Supplement 4

Supplement 5

Supplement 6

Supplement 7
