# Supplementary material for: Establishing MS2-MCP-based single-molecule RNA visualization in Schizosaccharomyces pombe
Source: bioRxiv. 2026 Mar 9:2026.03.09.710516. Preprint. [Version 1] doi: 10.64898/2026.03.09.710516 (PMC13061047; doi:10.64898/2026.03.09.710516)
Supplement: Supplement 1 [file media-1.pdf]

Figure S1

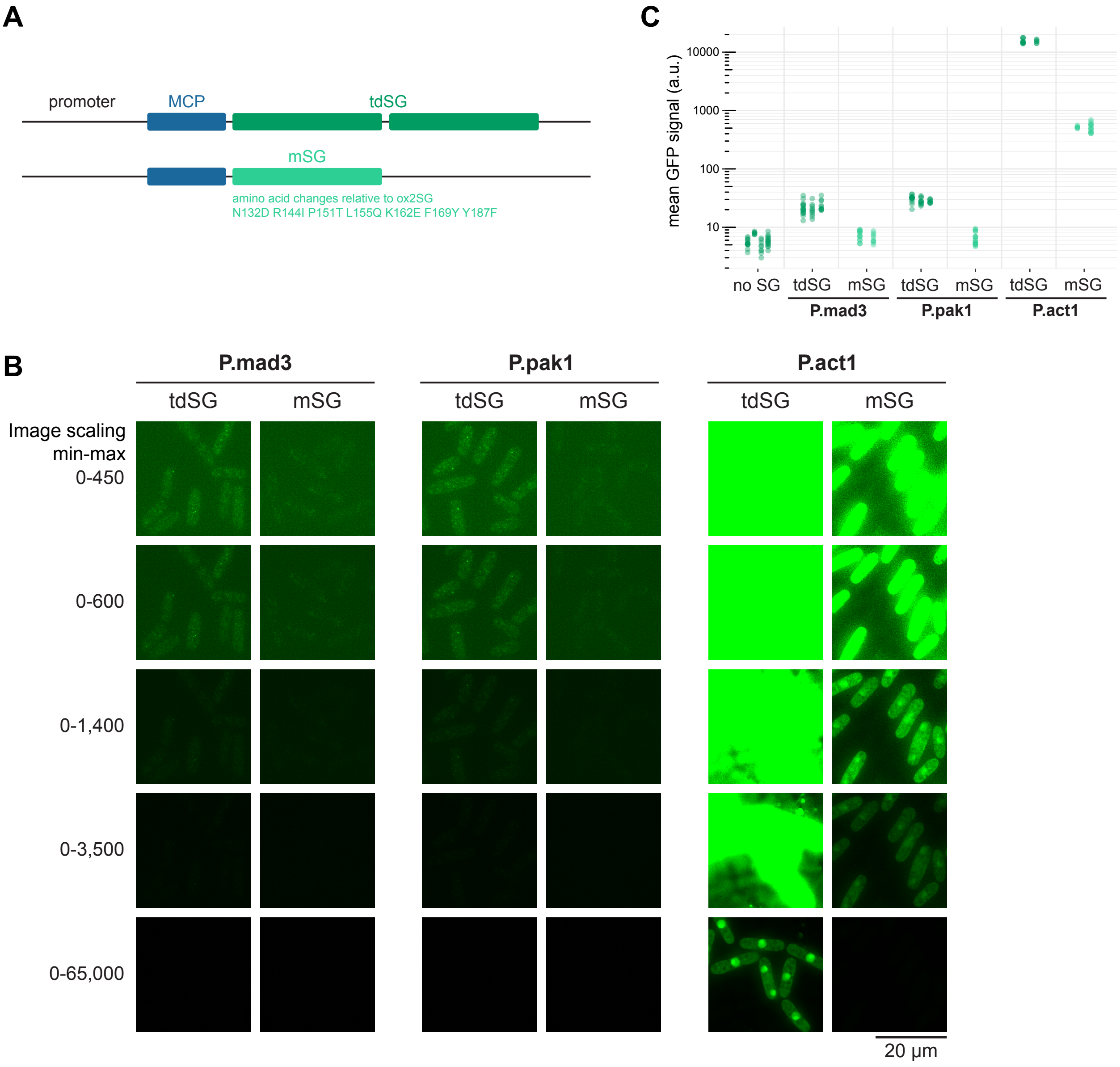

Figure S1. Comparison between tandem and monomeric StayGold fused to MCP.
(A) Schematic showing MCP-NLS-td8ox2SG (tandem SG, tdSG) and MCP-NLS-mSG (monomeric SG, mSG); mSG carries 7 amino acid changes relative to ox2SG. **(B)** Example images from strains expressing *mad2*-24xMS2 and MCP-NLS-tdSG or MCP-NLS-mSG from the *mad3*, *pak1*, or *act1* promoter. Image acquisition conditions were the same; each field of view is shown using five different scaling settings in order to capture the breadth of signal intensities. Example images for tdSG are the same as in Fig. 1. **(C)** The mean StayGold signal intensity in single cells was quantified; dots are individual cells, columns are technical or biological replicates. Note that data are displayed on a logarithmic scale. Signal intensity obtained from mSG is considerably less than half of that obtained from tdSG.

Figure S2

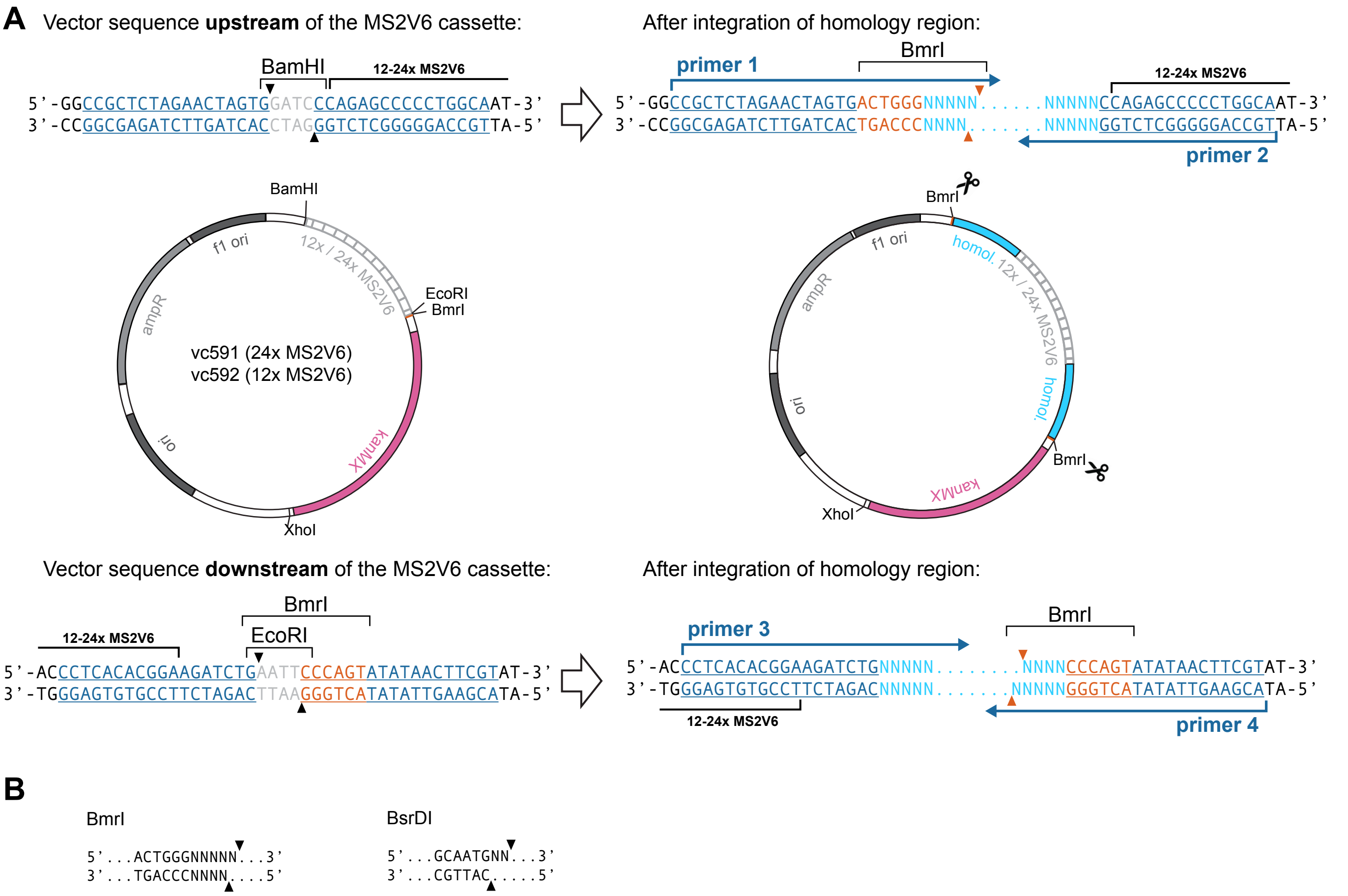

Figure S2. Attaching homology regions to MS2V6 repeats.
(A) Strategy for the integration of upstream and downstream homology regions (cyan), when BmrI is used as enzyme to cut out the piece to be transformed. The vectors already contain a BmrI site that overlaps with the EcoRI site downstream of the MS2V6 repeats. The upstream homology region is amplified with primers 1 and 2, the downstream homology region with primers 3 and 4. The PCR fragments are integrated into the BamHI/EcoRI-digested vector by Gibson assembly. Sequence regions: dark blue and underlined, regions of overlap with the vector for Gibson assembly; gray, nucleotides that become removed during Gibson assembly; orange, BmrI recognition site; cyan, inserted homology region; arrowheads: cut sites. In situations where a BmrI site is present in one of the homology regions, BsrDI sites can be introduced instead. **(B)** Type IIS restriction enzymes, such as BmrI and BsrDI, allow cleaving out the homology–MS2V6–homology fragment without leaving any traces of exogenous sequence.

Figure S3

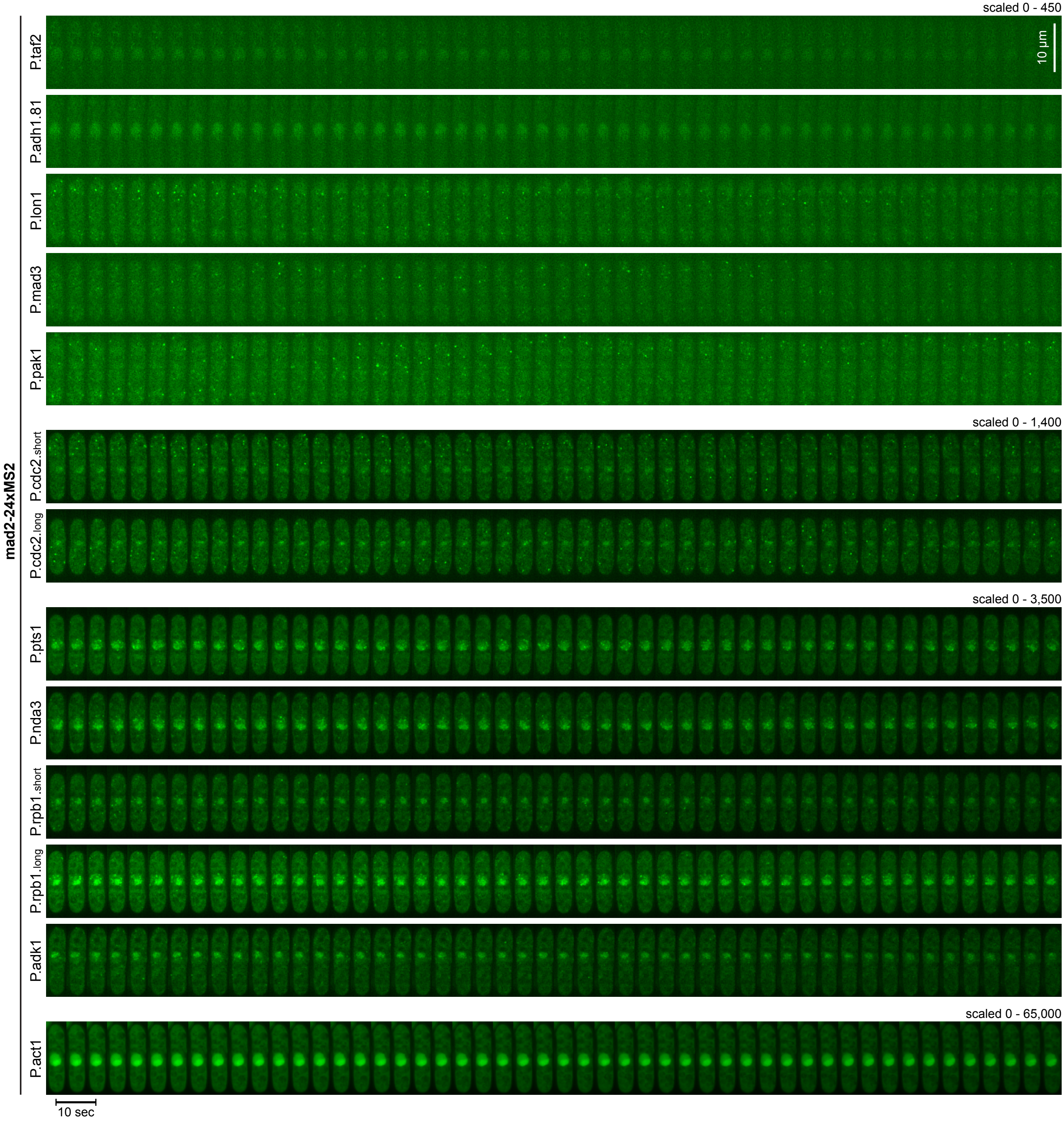

Figure S3. Overview of all promoter-MCP-tdSG combinations tested for *mad2*-24xMS2 mRNA imaging.
Kymographs from live-cell imaging of the indicated strains expressing *mad2*-24xMS2. MCP-tdSG was expressed from the indicated promoters. Images are maximum intensity projections of the Z-stack. Note the different scaling settings for displaying the images, necessitated by the different expression levels.

Figure S4

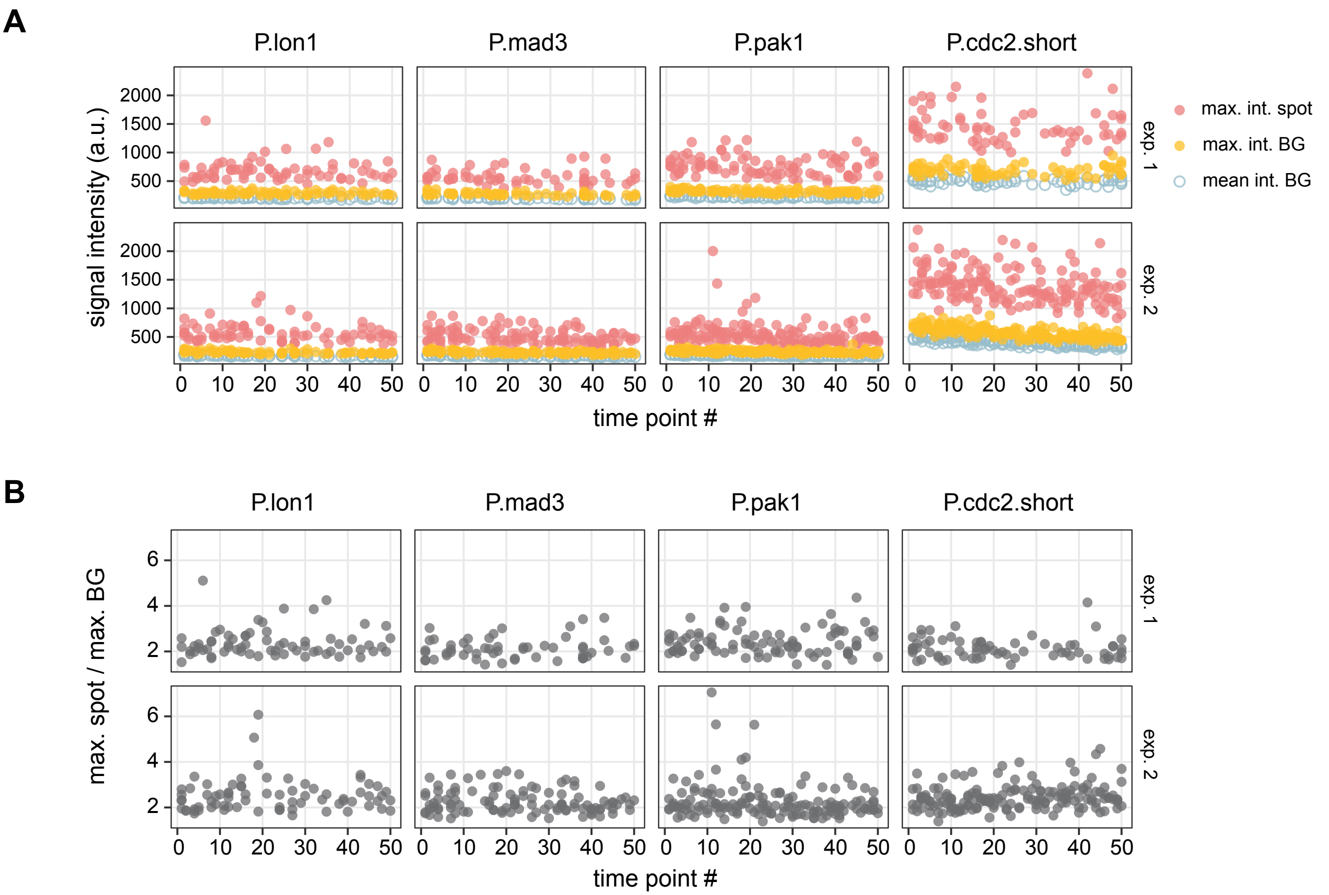

Figure S4. Quantification of cytoplasmic RNA signals.
Quantification of cytoplasmic dot-like signals (spots) and cytoplasmic background (BG) over time from two different experiments (exp. 1, exp. 2). Time points are spaced by 5 sec. **(A)** The maximum signal intensity is plotted for spots (salmon) and cytoplasmic background (yellow); the mean intensity for the cytoplasmic background is shown in addition (cyan circles). **(B)** Ratio between the maximum intensity of a spot and the maximum intensity of the cytoplasmic background in the same cell. The number of quantified spots per experiment and strain ranged from 56 to 90 for exp. 1 and 74 to 162 for exp. 2.

Figure S5

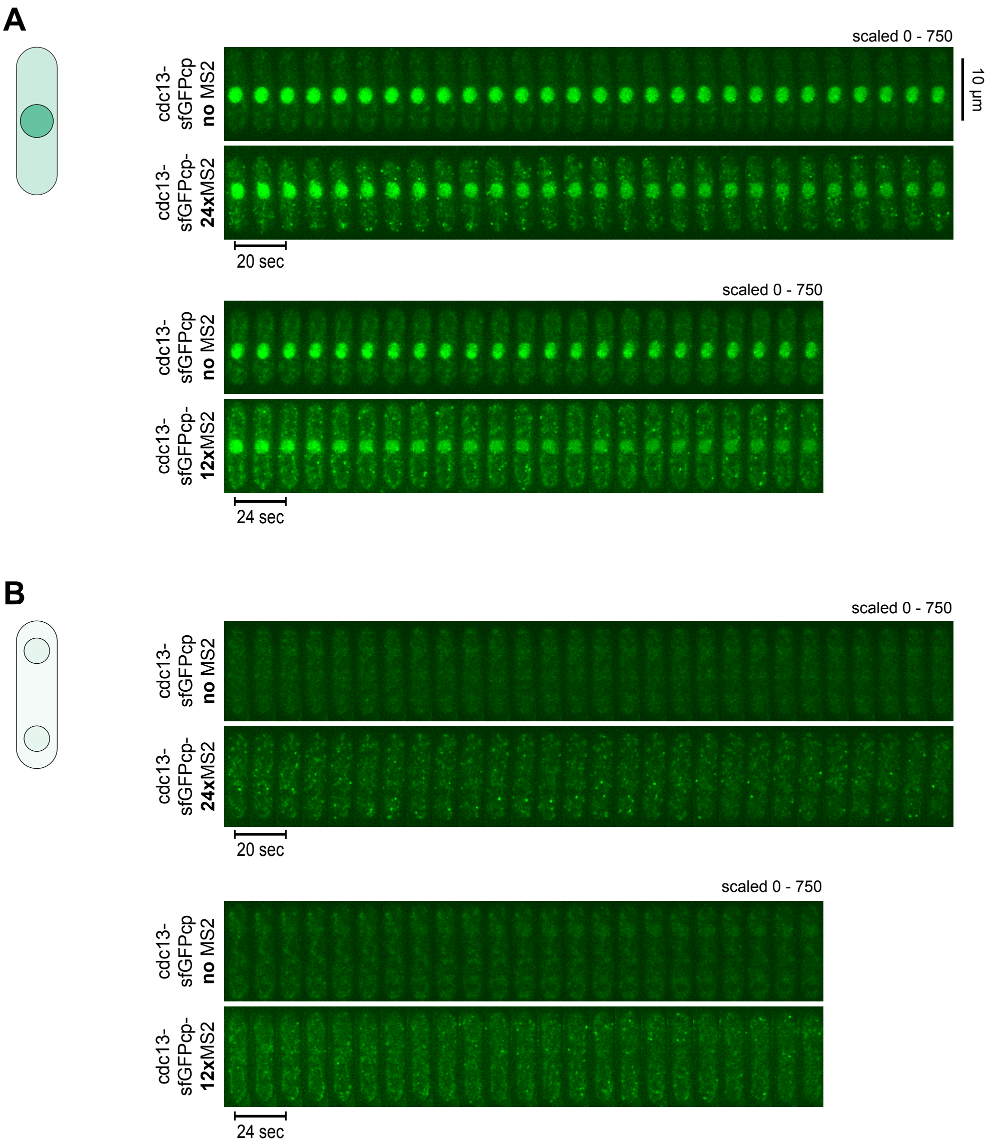

Figure S5. Additional representative kymographs from cells expressing *cdc13*-sfGFPcp-MS2 and P.mad3-MCP-tdSG. Kymographs from live-cell imaging of the indicated strains. A strain without integration of MS2 repeats is shown as control. Images were recorded every 5 sec for *cdc13*-sfGFPcp-24xMS2 and every 6 sec for *cdc13*-sfGFPcp-12xMS2; only every second image (every 10 sec and 12 sec, respectively) is shown. Images are maximum intensity projections of the Z-stack. **(A)** Cells in interphase with nuclear Cdc13-sfGFPcp signal. **(B)** Cells after nuclear division when the Cdc13 protein has been degraded.

Figure S6

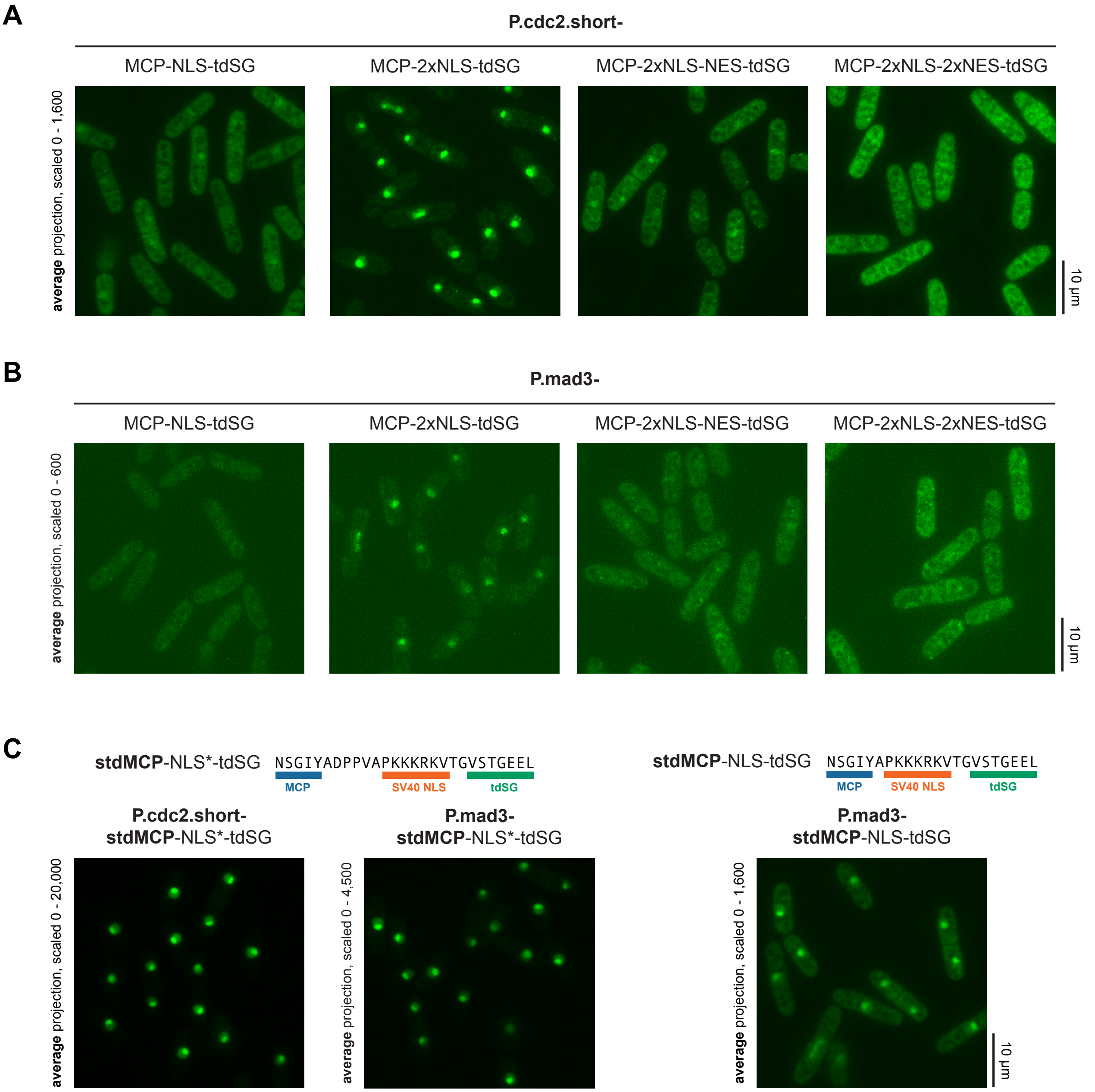

Figure S6. Additional representative images for strains with different NLS/NES combinations. Representative overview images of the indicated strains expressing *mad2*-24xMS2 and MCP-tdSG with different NLSs or NLS/NES combinations. Shown are average intensity projections (not maximum intensity projections) to adequately represent the nucleo-cytoplasmic ratios. Note the different scaling settings for displaying the images, necessitated by the different expression levels and nuclear enrichment. **(A)** Cells expressing MCP-tdSG from the short version of the *cdc2* promoter. **(B)** Cells expressing MCP-tdSG from the *mad3* promoter. **(C)** Cells expressing synonymized tandem MCP (stdMCP) with either a longer (NLS*) or a shorter (NLS) linker between MCP and NLS.

Table S1. ***S. pombe*** **strains.**

Name	Mating type	Genotype	Figure(s)	Simplified genotype
SX293	*h-*	*leu1- ura4+::P.taf2(244-1)-MCP-NLS-td8ox2StayGold-Scer\T.ADH1 mad2-ntG606A-ymEGFP-Y66L-3UTR.mad2-G0G-24xMS2V6*	1, S3	*P.taf2-MCP-NLS-tdSG mad2-24xMS2*
SX291	*h-*	*leu1- ura4+::P.lon1(451-1)-MCP-NLS-td8ox2StayGold-Scer\T.ADH1 mad2-ntG606A-ymEGFP-Y66L-3UTR.mad2-G0G-24xMS2V6*	1, 3, S4	*P.lon1-MCP-NLS-tdSG mad2-24xMS2*
SX258	*?*	*leu1- ura4+::P.mad3(717-1)-MCP-NLS-td8ox2StayGold-Scer\T.ADH1 mad2-ntG606A-ymEGFP-Y66L-3UTR.mad2-G0G-24xMS2V6*	1, 3, S1, S4, S6	*P.mad3-MCP-NLS-tdSG mad2-24xMS2*
SX716	*h-*	*leu1- ura4+::P.pak1-MCP-NLS-td8ox2StayGold-Scer\T.ADH1 mad2-ntG606A-ymEGFP-Y66L-3UTR.mad2-G0G-24xMS2V6*	1, 3, S1, S4	*P.pak1-MCP-NLS-tdSG mad2-24xMS2*
SX717	*h-*	*leu1- ura4+::P.adh1.81-MCP-NLS-td8ox2StayGold-Scer\T.ADH1 mad2-ntG606A-ymEGFP-Y66L-3UTR.mad2-G0G-24xMS2V6*	1, S3	*P.adh1.81-MCP-NLS-tdSG mad2-24xMS2*
SX296	*h-*	*leu1- ura4+::P.cdc2(355-1)-MCP-NLS-td8ox2StayGold-Scer\T.ADH1 mad2-ntG606A-ymEGFP-Y66L-3UTR.mad2-G0G-24xMS2V6*	1, 3, 5, S4, S6	*P.cdc2.short-MCP-NLS-tdSG mad2-24xMS2*
SX297	*h-*	*leu1- ura4+::P.cdc2(913-1)-MCP-NLS-td8ox2StayGold-Scer\T.ADH1 mad2-ntG606A-ymEGFP-Y66L-3UTR.mad2-G0G-24xMS2V6*	1, S3	*P.cdc2.long-MCP-NLS-tdSG mad2-24xMS2*
SX292	*h-*	*leu1- ura4+::P.pts1(378-1)-MCP-NLS-td8ox2StayGold-Scer\T.ADH1 mad2-ntG606A-ymEGFP-Y66L-3UTR.mad2-G0G-24xMS2V6*	1, S3	*P.pts1-MCP-NLS-tdSG mad2-24xMS2*
SX283	*h-*	*leu1- ura4+::P.nda3(620-1)-MCP-NLS-td8ox2StayGold-Scer\T.ADH1 mad2-ntG606A-ymEGFP-Y66L-3UTR.mad2-G0G-24xMS2V6*	1, S3	*P.nda3-MCP-NLS-tdSG mad2-24xMS2*
SX284	*h-*	*leu1- ura4+::P.rpb1(214-1)-MCP-NLS-td8ox2StayGold-Scer\T.ADH1 mad2-ntG606A-ymEGFP-Y66L-3UTR.mad2-G0G-24xMS2V6*	1, S3	*P.rpb1.short-MCP-NLS-tdSG mad2-24xMS2*
SX285	*h-*	*leu1- ura4+::P.rpb1(647-1)-MCP-NLS-td8ox2StayGold-Scer\T.ADH1 mad2-ntG606A-ymEGFP-Y66L-3UTR.mad2-G0G-24xMS2V6*	1, S3	*P.rpb1.long-MCP-NLS-tdSG mad2-24xMS2*
SX282	*h-*	*leu1- ura4+::P.adk1(338-1)-MCP-NLS-td8ox2StayGold-Scer\T.ADH1 mad2-ntG606A-ymEGFP-Y66L-3UTR.mad2-G0G-24xMS2V6*	1, S3	*P.adk1-MCP-NLS-tdSG mad2-24xMS2*
SX250	*?*	*leu1- ura4+::P.act1(822-1)-MCP-NLS-td8ox2StayGold-Scer\T.ADH1 mad2-ntG606A-ymEGFP-Y66L-3UTR.mad2-G0G-24xMS2V6*	1, S1, S3	*P.act1-MCP-NLS-tdSG mad2-24xMS2*
SX257	*?*	*leu1- ura4+::P.mad3(717-1)-MCP-NLS-mStayGold-Scer\T.ADH1 mad2-ntG606A-ymEGFP-Y66L-3UTR.mad2-G0G-24xMS2V6*	S1	*P.mad3-MCP-NLS-mSG mad2-24xMS2*
SX259	*?*	*leu1- ura4+::P.pak1(630-1)-MCP-NLS-mStayGold-Scer\T.ADH1 mad2-ntG606A-ymEGFP-Y66L-3UTR.mad2-G0G-24xMS2V6*	S1	*P.pak1-MCP-NLS-mSG mad2-24xMS2*
SX249	*?*	*leu1- ura4+::P.act1(822-1)-MCP-mStayGold-Scer\T.ADH1 mad2-ntG606A-ymEGFP-Y66L-3UTR.mad2-G0G-24xMS2V6*	S1	*P.act1-MCP-NLS-mSG mad2-24xMS2*
SX279	*?*	*leu1- ura4-D18 mad2-ntG606A-ymEGFP-Y66L-3UTR.mad2-G0G-24xMS2V6*	3, S1	*mad2-24xMS2*
SX280	*?*	*leu1- ura4-D18 mad2-ntG606A-ymEGFP-Y66L-3UTR.mad2-G0G-12xMS2V6*	S1	*mad2-12xMS2*

SX724	*h-*	*leu1- ura4+::P.lon1-MCP-NLS-td8ox2StayGold-Scer\T.ADH1*	3	*P.lon1-MCP-NLS-tdSG*
SX253	*h-*	*leu1- ura4+::P.mad3(717-1)-MCP-NLS-td8ox2StayGold-Scer\T.ADH1*	3	*P.mad3-MCP-NLS-tdSG*
SX725	*h-*	*leu1- ura4+::P.pak1-MCP-NLS-td8ox2StayGold-Scer\T.ADH1*	3	*P.pak1-MCP-NLS-tdSG*
SX723	*h-*	*leu1- ura4+::P.cdc2(355-1)-MCP-NLS-td8ox2StayGold-Scer\T.ADH1*	3	*P.cdc2.short-MCP-NLS-tdSG*
SX761	*h-*	*leu1-32::P.cdc13-cdc13-S177S-sfGFPcp:leu1+ ura4+::P.mad3(717-1)-MCP-NLS-td8ox2StayGold-Scer\T.ADH1*	3, S5	*P.mad3-MCP-NLS-mSG cdc13-sfGFPcp* (exogenous)
SX289	*h-*	*leu1-32::P.cdc13-cdc13-S177S-sfGFPcp-3UTR.cdc13-C332C-24xMS2V6:leu1+ ura4+::P.mad3(717-1)-MCP-NLS-td8ox2StayGold-Scer\T.ADH1*	3, S5	*P.mad3-MCP-NLS-mSG cdc13-sfGFPcp-24xMS2(332)* (exogenous)
SX287	*h-*	*leu1-32::P.cdc13-cdc13-S177S-sfGFPcp-3UTR.cdc13-C332C-12xMS2V6:leu1+ ura4+::P.mad3(717-1)-MCP-NLS-td8ox2StayGold-Scer\T.ADH1*	3, S5	*P.mad3-MCP-NLS-mSG cdc13-sfGFPcp-12xMS2(332)* (exogenous)
SX286	*h-*	*leu1-32::P.cdc13-cdc13-S177S-sfGFPcp-3UTR.cdc13-A205A-12xMS2V6:leu1+ ura4+::P.mad3(717-1)-MCP-NLS-td8ox2StayGold-Scer\T.ADH1*	3, S5	*P.mad3-MCP-NLS-mSG cdc13-sfGFPcp-12xMS2(205)* (exogenous)
SX288	*h-*	*leu1-32::P.cdc13-cdc13-S177S-sfGFPcp-3UTR.cdc13-A205A-24xMS2V6:leu1+ ura4+::P.mad3(717-1)-MCP-NLS-td8ox2StayGold-Scer\T.ADH1*	S5	*P.mad3-MCP-NLS-mSG cdc13-sfGFPcp-24xMS2(205)* (exogenous)
SX720	*h-*	*leu1- ura4+::P.cdc2(355-1)-MCP-2xNLS-td8ox2StayGold-Scer\T.ADH1 mad2-ntG606A-ymEGFP-Y66L-3UTR.mad2-G0G-24xMS2V6*	4, S6	*P.cdc2.short-MCP-2xNLS-tdSG mad2-24xMS2*
SX721	*h-*	*leu1- ura4+::P.cdc2(355-1)-MCP-2xNLS-NES-td8ox2StayGold-Scer\T.ADH1 mad2-ntG606A-ymEGFP-Y66L-3UTR.mad2-G0G-24xMS2V6*	4, S6	*P.cdc2.short-MCP-2xNLS-NES-tdSG mad2-24xMS2*
SX722	*h-*	*leu1- ura4+::P.cdc2(355-1)-MCP-2xNLS-2xNES-td8ox2StayGold-Scer\T.ADH1 mad2-ntG606A-ymEGFP-Y66L-3UTR.mad2-G0G-24xMS2V6*	4, S6	*P.cdc2.short-MCP-2xNLS-2xNES-tdSG mad2-24xMS2*
SX719	*h-*	*leu1- ura4+::P.cdc2(355-1)-stdMCP-NLS-td8ox2StayGold-Scer\T.ADH1 mad2-ntG606A-ymEGFP-Y66L-3UTR.mad2-G0G-24xMS2V6*	4, S6	*P.cdc2.short-stdMCP-NLS*-tdSG mad2-24xMS2*
SX712	*h-*	*leu1- ura4+::P.mad3(717-1)-MCP-2xNLS-td8ox2StayGold-Scer\T.ADH1 mad2-ntG606A-ymEGFP-Y66L-3UTR.mad2-G0G-24xMS2V6*	S6	*P.mad3-MCP-2xNLS-tdSG mad2-24xMS2*
SX733	*h-*	*leu1- ura4+::P.mad3-MCP-2xNLS-NES-td8ox2StayGold-Scer\T.ADH1 mad2-ntG606A-ymEGFP-Y66L-3UTR.mad2-G0G-24xMS2V6*	S6	*P.mad3-MCP-2xNLS-NES-tdSG mad2-24xMS2*
SX714	*h-*	*leu1- ura4+::P.mad3(717-1)-MCP-2xNLS-2xNES-td8ox2StayGold-Scer\T.ADH1 mad2-ntG606A-ymEGFP-Y66L-3UTR.mad2-G0G-24xMS2V6*	S6	*P.mad3-MCP-2xNLS-2xNES-tdSG mad2-24xMS2*
SX718	*h-*	*leu1- ura4+::P.mad3(717-1)-stdMCP-NLS-td8ox2StayGold-Scer\T.ADH1 mad2-ntG606A-ymEGFP-Y66L-3UTR.mad2-G0G-24xMS2V6*	S6	*P.mad3-stdMCP-NLS*-tdSG mad2-24xMS2*
SX767	*?*	*leu1- mad2-ntG606A-ymEGFP-Y66L-3UTR.mad2-G0G-24xMS2V6 ura4+::P.mad3(717-1)-stdMCP-NLS-td8ox2StayGold-Scer\T.ADH1*	S6	*P.mad3-stdMCP-NLS-tdSG mad2-24xMS2*

Table S2. Vectors.

ID	name	insert	notes	origin	to be deposited
vc547	pUra4-P.mad3-MCP-NLS-mSG	P.mad3(717-1)-MCP-NLS-mStayGold-Scer\T.ADH1		derived from pUra4AfeI, Addgene 133467, by Aleksandar Vještica, Sophie Martin, et al., doi: 10.1242/jcs.240754	N
vc548	pUra4-P.mad3-MCP-NLS-tdSG	P.mad3(717-1)-MCP-NLS-td8ox2StayGold-Scer\T.ADH1		derived from pUra4AfeI, Addgene 133467, by Aleksandar Vještica, Sophie Martin, et al., doi: 10.1242/jcs.240754	Y
vc549	pUra4-P.pak1-MCP-NLS-mSG	P.pak1(630-1)-MCP-NLS-mStayGold-Scer\T.ADH1		derived from pUra4AfeI, Addgene 133467, by Aleksandar Vještica, Sophie Martin, et al., doi: 10.1242/jcs.240754	N
vc582	pUra4-P.act1-MCP-mSG	P.act1(822-1)-MCP-GGGGS-mStayGold-Scer\T.ADH1		derived from pUra4AfeI, Addgene 133467, by Aleksandar Vještica, Sophie Martin, et al., doi: 10.1242/jcs.240754	N
vc583	pUra4-P.act1-MCP-NLS-tdSG	P.act1(822-1)-MCP-NLS-td8ox2StayGold-Scer\T.ADH1		derived from pUra4AfeI, Addgene 133467, by Aleksandar Vještica, Sophie Martin, et al., doi: 10.1242/jcs.240754	N
vc591	pET264-pUC_24xMS2V6_homIns	24xMS2V6		slightly modified version of Addgene 104393 by Evelina Tutucci and Robert Singer, doi: 10.1038/nmeth.4502	Y
vc592	pET251-pUC_12xMS2V6_homIns	12xMS2V6		slightly modified version of Addgene 104392 by Evelina Tutucci and Robert Singer, doi: 10.1038/nmeth.4502	Y
vc603	pUra4-P.adk1-MCP-NLS-tdSG	P.adk1(338-1)-MCP-NLS-td8ox2StayGold-Scer\T.ADH1		derived from pUra4AfeI, Addgene 133467, by Aleksandar Vještica, Sophie Martin, et al., doi: 10.1242/jcs.240754	N
vc604	pUra4-P.nda3-MCP-NLS-tdSG	P.nda3(620-1)-MCP-NLS-td8ox2StayGold-Scer\T.ADH1		derived from pUra4AfeI, Addgene 133467, by Aleksandar Vještica, Sophie Martin, et al., doi: 10.1242/jcs.240754	N
vc605	pUra4-P.rpb1.short-MCP-NLS-tdSG	P.rpb1(214-1)-MCP-NLS-td8ox2StayGold-Scer\T.ADH1		derived from pUra4AfeI, Addgene 133467, by Aleksandar Vještica, Sophie Martin, et al., doi: 10.1242/jcs.240754	N
vc606	pUra4-P.rpb1.long-MCP-NLS-tdSG	P.rpb1(647-1)-MCP-NLS-td8ox2StayGold-Scer\T.ADH1		derived from pUra4AfeI, Addgene 133467, by Aleksandar Vještica, Sophie Martin, et al., doi: 10.1242/jcs.240754	N
vc607	pDUAL-cdc13-sfGFP-12xMS2V6_3p-205	P.cdc13-cdc13-sfGFPcp-12xMS2V6 (pos. 205 in 3' UTR)		derived from pDUAL, Matsuyama, Yoshida et al., doi: 10.1002/yea.1181	N

ID	name	insert	notes	origin	to be deposited
vc608	pDUAL-cdc13-sfGFP-12xMS2V6_3p-332	P.cdc13-cdc13-sfGFPcp-12xMS2V6 (pos. 332 in 3' UTR)		derived from pDUAL, Matsuyama, Yoshida et al., doi: 10.1002/yea.1181	N
vc609	pDUAL-cdc13-sfGFP-24xMS2V6_3p-305	P.cdc13-cdc13-sfGFPcp-24xMS2V6 (pos. 205 in 3' UTR)		derived from pDUAL, Matsuyama, Yoshida et al., doi: 10.1002/yea.1181	N
vc610	pDUAL-cdc13-sfGFP-24xMS2V6_3p-332	P.cdc13-cdc13-sfGFPcp-24xMS2V6 (pos. 332 in 3' UTR)		derived from pDUAL, Matsuyama, Yoshida et al., doi: 10.1002/yea.1181	N
vc617	pUra4-P.cdc2.short-MCP-NLS-tdSG	P.cdc2(355-1)-MCP-NLS-td8ox2StayGold-Scer\T.ADH1	requires limited digest since AfeI site present in cdc2 promoter	derived from pUra4AfeI, Addgene 133467, by Aleksandar Vještica, Sophie Martin, et al., doi: 10.1242/jcs.240754	N
vc618	pUra4-P.cdc2.long-MCP-NLS-tdSG	P.cdc2(913-1)-MCP-NLS-td8ox2StayGold-Scer\T.ADH1	requires limited digest since AfeI site present in cdc2 promoter	derived from pUra4AfeI, Addgene 133467, by Aleksandar Vještica, Sophie Martin, et al., doi: 10.1242/jcs.240754	N
vc619	pUra4-P.lon1-MCP-NLS-tdSG	P.lon1(451-1)-MCP-NLS-td8ox2StayGold-Scer\T.ADH1		derived from pUra4AfeI, Addgene 133467, by Aleksandar Vještica, Sophie Martin, et al., doi: 10.1242/jcs.240754	Y
vc620	pUra4-P.pts1-MCP-NLS-tdSG	P.pts1(378-1)-MCP-NLS-td8ox2StayGold-Scer\T.ADH1		derived from pUra4AfeI, Addgene 133467, by Aleksandar Vještica, Sophie Martin, et al., doi: 10.1242/jcs.240754	N
vc621	pUra4-P.taf2-MCP-NLS-tdSG	P.taf2(244-1)-MCP-NLS-td8ox2StayGold-Scer\T.ADH1		derived from pUra4AfeI, Addgene 133467, by Aleksandar Vještica, Sophie Martin, et al., doi: 10.1242/jcs.240754	N
vc633	pUra4-P.pak1-MCP-NLS-tdSG	P.pak1(630-1)-MCP-NLS-td8ox2StayGold-Scer\T.ADH1	pak1 is an alternative gene name for shk1	derived from pUra4AfeI, Addgene 133467, by Aleksandar Vještica, Sophie Martin, et al., doi: 10.1242/jcs.240754	Y
vc634	pUra4-P.adh1.81-MCP-NLS-tdSG	P.adh1.81-MCP-NLS-td8ox2StayGold-Scer\T.ADH1		derived from pUra4AfeI, Addgene 133467, by Aleksandar Vještica, Sophie Martin, et al., doi: 10.1242/jcs.240754	N
vc640	pUra4-P.mad3-MCP-2xNLS-tdSG	P.mad3(717-1)-MCP-2xNLS-td8ox2StayGold-Scer\T.ADH1		derived from pUra4AfeI, Addgene 133467, by Aleksandar Vještica, Sophie Martin, et al., doi: 10.1242/jcs.240754	N
vc641	pUra4-P.mad3-MCP-2xNLS-NES-tdSG	P.mad3(717-1)-MCP-2xNLS-NES-td8ox2StayGold-Scer\T.ADH1		derived from pUra4AfeI, Addgene 133467, by Aleksandar Vještica, Sophie Martin, et al., doi: 10.1242/jcs.240754	Y

ID	name	insert	notes	origin	to be deposited
vc642	pUra4-P.mad3-MCP-2xNLS-2xNES-tdSG	P.mad3(717-1)-MCP-2xNLS-2xNES-td8ox2StayGold-Scer\T.ADH1		derived from pUra4AfeI, Addgene 133467, by Aleksandar Vještica, Sophie Martin, et al., doi: 10.1242/jcs.240754	Y
vc643	pUra4-P.cdc2.short-MCP-2xNLS-tdSG	P.cdc2(355-1)-MCP-2xNLS-td8ox2StayGold-Scer\T.ADH1	requires limited digest since AfeI site present in cdc2 promoter	derived from pUra4AfeI, Addgene 133467, by Aleksandar Vještica, Sophie Martin, et al., doi: 10.1242/jcs.240754	N
vc644	pUra4-P.cdc2.short-MCP-2xNLS-NES-tdSG	P.cdc2(355-1)-MCP-2xNLS-NES-td8ox2StayGold-Scer\T.ADH1	requires limited digest since AfeI site present in cdc2 promoter	derived from pUra4AfeI, Addgene 133467, by Aleksandar Vještica, Sophie Martin, et al., doi: 10.1242/jcs.240754	Y
vc645	pUra4-P.cdc2.short-MCP-2xNLS-2xNES-tdSG	P.cdc2(355-1)-MCP-2xNLS-2xNES-td8ox2StayGold-Scer\T.ADH1	requires limited digest since AfeI site present in cdc2 promoter	derived from pUra4AfeI, Addgene 133467, by Aleksandar Vještica, Sophie Martin, et al., doi: 10.1242/jcs.240754	Y
vc646	pUra4-P.mad3-stdMCP-NLS*-tdSG	P.mad3(717-1)-stdMCP-NLS*-td8ox2StayGold-Scer\T.ADH1		derived from pUra4AfeI, Addgene 133467, by Aleksandar Vještica, Sophie Martin, et al., doi: 10.1242/jcs.240754; stdMCP from Addgene 98916 by Bin Wu, Robert Singer, et al., doi: 10.1101/gad.259358.115; SV40 NLS with N-terminal linker	N
vc647	pUra4-P.cdc2.short-stdMCP-NLS*-tdSG	P.cdc2(355-1)-stdMCP-NLS*-td8ox2StayGold-Scer\T.ADH1	requires limited digest since AfeI site present in cdc2 promoter	derived from pUra4AfeI, Addgene 133467, by Aleksandar Vještica, Sophie Martin, et al., doi: 10.1242/jcs.240754; stdMCP from Addgene 98916 by Bin Wu, Robert Singer, et al., doi: 10.1101/gad.259358.115; SV40 NLS with N-terminal linker	N
vc655	pUra4-P.cdc2.short(AfeImut)-MCP-NLS-tdSG	P.cdc2(355-1,AfeImut)-MCP-NLS-td8ox2StayGold-Scer\T.ADH1	AfeI site within cdc2 promoter mutated by removing one nucleotide	derived from pUra4AfeI, Addgene 133467, by Aleksandar Vještica, Sophie Martin, et al., doi: 10.1242/jcs.240754	Y
vc669	pUra4-P.mad3-stdMCP-NLS-tdSG	P.mad3(717-1)-stdMCP-NLS-td8ox2StayGold-Scer\T.ADH1		derived from pUra4AfeI, Addgene 133467, by Aleksandar Vještica, Sophie Martin, et al., doi: 10.1242/jcs.240754; stdMCP from Addgene 98916 by Bin Wu, Robert Singer, et al., doi: 10.1101/gad.259358.115	Y
