# Supplementary figures and images for: Establishing MS2-MCP-based single-molecule RNA visualization in Schizosaccharomyces pombe
Source: bioRxiv. 2026 Mar 9:2026.03.09.710516. Preprint. [Version 1] doi: 10.64898/2026.03.09.710516 (PMC13061047; doi:10.64898/2026.03.09.710516)

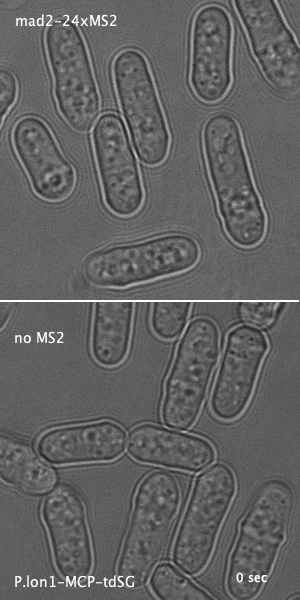

Supplement: Supplement 2 [file media-2.gif]

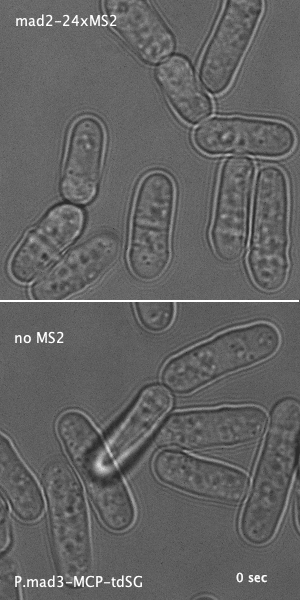

Supplement: Supplement 3 [file media-3.gif]

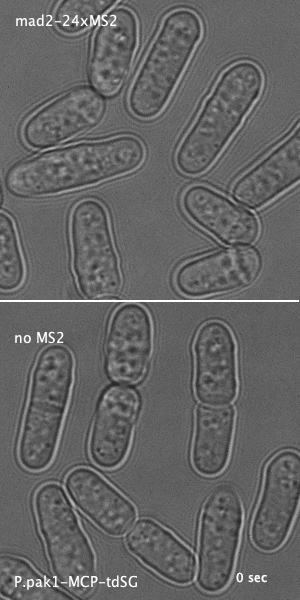

Supplement: Supplement 4 [file media-4.gif]

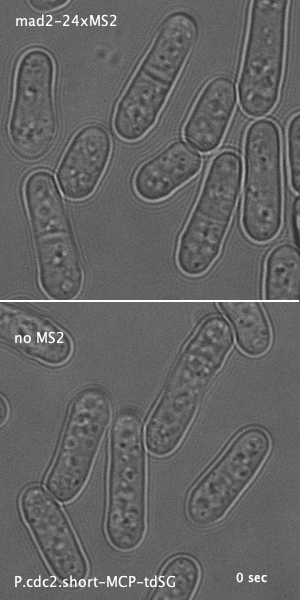

Supplement: Supplement 5 [file media-5.gif]

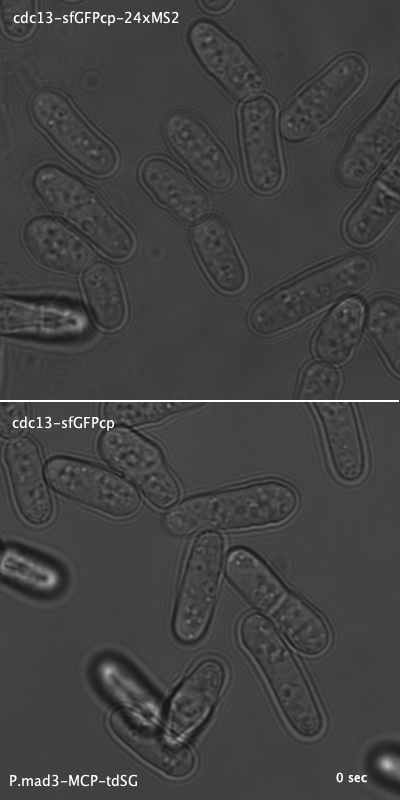

Supplement: Supplement 6 [file media-6.gif]

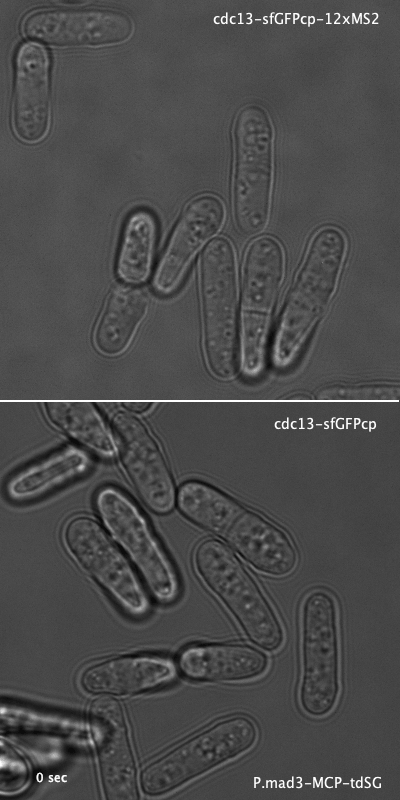

Supplement: Supplement 7 [file media-7.gif]
